# Quantitative Expression Analysis in *Brassica napus* by Northern Blot Analysis and Reverse Transcription-Quantitative PCR in a Complex Experimental Setting

**DOI:** 10.1371/journal.pone.0163679

**Published:** 2016-09-29

**Authors:** Annekathrin Rumlow, Els Keunen, Jan Klein, Philip Pallmann, Anja Riemenschneider, Ann Cuypers, Jutta Papenbrock

**Affiliations:** 1 Institute of Botany, Leibniz University Hannover, Hannover, Germany; 2 Centre for Environmental Sciences, Hasselt University, Diepenbeek, Belgium; 3 Department of Mathematics and Statistics, Lancaster University, Lancaster, United Kingdom; Universidade de Lisboa Instituto Superior de Agronomia, PORTUGAL

## Abstract

Analysis of gene expression is one of the major ways to better understand plant reactions to changes in environmental conditions. The comparison of many different factors influencing plant growth challenges the gene expression analysis for specific gene-targeted experiments, especially with regard to the choice of suitable reference genes. The aim of this study is to compare expression results obtained by Northern blot, semi-quantitative PCR and RT-qPCR, and to identify a reliable set of reference genes for oilseed rape (*Brassica napus* L.) suitable for comparing gene expression under complex experimental conditions. We investigated the influence of several factors such as sulfur deficiency, different time points during the day, varying light conditions, and their interaction on gene expression in oilseed rape plants. The expression of selected reference genes was indeed influenced under these conditions in different ways. Therefore, a recently developed algorithm, called GrayNorm, was applied to validate a set of reference genes for normalizing results obtained by Northern blot analysis. After careful comparison of the three methods mentioned above, Northern blot analysis seems to be a reliable and cost-effective alternative for gene expression analysis under a complex growth regime. For using this method in a quantitative way a number of references was validated revealing that for our experiment a set of three references provides an appropriate normalization. Semi-quantitative PCR was prone to many handling errors and difficult to control while RT-qPCR was very sensitive to expression fluctuations of the reference genes.

## Introduction

In recent years, different techniques and new methods have been developed to investigate the reaction of plants and their underlying regulating mechanisms to different environmental changes at the level of gene expression. Depending on the research question, different methods have been applied to analyze the expression levels of genes and in this way measure the abundance of specific mRNA transcripts. Northern blot analysis [1] was often used as the gold standard to estimate the expression level of a gene by visualizing the abundance of its mRNA transcript in a sample. During the hybridization step, a labeled probe is required to form a double-stranded RNA-DNA or RNA-RNA molecule that can be detected by using an antibody-assisted technology in a colorimetric or a luminescent reaction. The resulting intensity of the color or light signal is proportional to the enzyme activity, which in turn is correlated to the transcript level of the gene of interest (GOI). Thus, expression analysis with this method is focused on an endpoint signal. Northern blot analysis is the only method providing information on transcript size and integrity of the isolated RNA. Furthermore, it is a very versatile method including different labeling and detection methods and acceptance of different lengths of probes. One advantage is that RNA quantity and quality can be verified after gel electrophoresis, which makes it possible to evaluate the progress. On the other hand, there are a number of disadvantages such as the need for high quality RNA in large amounts. In addition, intensive washing steps, followed by a detection step of hybridization products make the Northern blot technique time-consuming.

Due to the discovery of the reverse transcriptase (RT) enzyme converting RNA into DNA, very low GOI transcript levels can be detected with a high specificity using PCR-based methods. To quantify the transcript levels of a target gene, semi-quantitative non-real-time PCR (sqPCR) has been applied. After a defined number of cycles in the range of the exponential phase, the reaction is stopped and the product visualized by electrophoretic separation and staining. By measuring the intensity of the band corresponding to the amplified product, the GOI expression level can be determined. However, this method has a number of pitfalls such as having to determine the cycle number for all samples, potentially leading to false interpretation of results. Therefore, this technique is suitable for investigating differences in the linear phase, e.g. in knockout studies to illustrate expression versus no expression (for an overview, see [2]). Although, this method is generally only accepted in knockout studies it is still in use for analyzing gene expression in plants treated by different treatments [3,4].

Nowadays, quantitative or real-time PCR (RT-qPCR) seems to be the method of choice for a rapid and reliable quantification of mRNA transcripts (for an overview, see [2,5]). This technique combines the PCR chemistry with the use of fluorescent reporter molecules to assess the rate of amplicon accumulation during the exponential phase in the course of the reaction cycles. RT-qPCR constitutes an excellent combination of sensitivity, specificity and reproducibility in a relatively short period of time [6]. A number of published RT-qPCR data showed a lack of experimental details such as RNA quality and integrity or PCR efficiencies, making a critical evaluation of the quality of the results by the reader difficult [7]. Therefore, a guideline was established called Minimum Information for Publication of Quantitative Real-Time PCR Experiments (MIQE), providing authors with a list of information that should be reported for RT-qPCR experiments [5]. However, the accuracy of the expression analysis—whether performed by Northern blot analysis or RT-qPCR—crucially depends on the use of stably expressed reference genes. This is very important when it comes to RT-qPCR analysis where the transcript level of each target gene is normalized to the expression of a combination of at least three reference genes ideally correcting for technical errors, e.g. introduced during the RT step and affecting all genes similarly [8]. Algorithms such as geNorm [9] and Normfinder [10] can be used to identify a combination of stable reference genes under specific experimental conditions. Based on expression levels of the best performing reference genes, a sample-specific normalization factor is calculated. Evaluating candidate genes as suitable reference genes using such algorithms increased the accuracy of gene expression analysis in mammals, yeast and bacteria. However, a number of publications surveyed from 2009–2011 and 2012–2013 showed inadequate normalization leading to results the reader cannot rely on properly [7].

Regarding expression analysis in plants a lack of an appropriate validation of reference genes in the past led to the use of candidate genes for normalization turning out to be not a good choice either (reviewed in [11]). To avoid such incidents, a number of reference genes in plants were tested under different stress conditions and in different tissues, resulting in different recommendations of reference genes for each condition [12–16]. Depending on the material and experimental settings, one has to search for an appropriate combination of reference genes whose expression is minimally affected under the given conditions [17,18]. However, candidate reference genes should be (re)validated in each subsequent experiment [19]. Furthermore, not all variability can be removed by reference genes, even when appropriate procedures are applied. Indeed, reference genes always show small or larger expression changes between tissues or treatments, which potentially cause differences in the calculation of GOI transcript levels leading to false biological conclusions. With the GrayNorm algorithm, a method was developed to maximize data accuracy by selecting the optimal combination of reference genes for each particular experiment [19].

We describe the technical prerequisites to quantitatively analyze the expression of specific genes in experiments where oilseed rape (*Brassica napus* L.) plants were grown at different sulfur (S) supplies and analyzed over a day/night cycle and continuous light as well. When the plants are grown under constant conditions, the circadian rhythms persist and oscillate with an endogenous period close to 24 h. In their natural environment, the plants are exposed to environmental “zeitgebers” such as light and temperature. It entrains the endogenous organismal clock in each cell with the local time (reviewed in [20,21]). Since S is an essential macronutrient required to synthesize the amino acids cysteine and methionine as well as glutathione, phytochelatins, vitamins and cofactors, the dependence of the circadian clock on S supply is evaluated in this study. Expression levels of genes involved in the circadian rhythm and the S assimilation were therefore determined using Northern blot analysis. In the first part of this study, its reliability was verified by comparing it to PCR-based expression analyses. In addition to a detailed statistical comparison, factors like working hours, ease of handling and costs for each sample are taken into account when evaluating our results. In the second part, we show the importance of selecting reliable reference genes under complex experimental settings affecting the plants in multiple ways. Therefore, a suitable combination of reference genes was determined by the GrayNorm algorithm to normalize expression levels obtained by Northern blot analysis. Results were additionally compared to data normalized with only one reference gene to highlight the importance of an adequate normalization.

## Material and Methods

### Plant growth

Oilseed rape (*Brassica napus* L.) seeds of cultivar Genie were obtained from the Deutsche Saatveredelung AG (DSV) (Lippstadt, Germany). For experiments under circadian and diurnal conditions, the seeds were germinated in a pot with a diameter of 8 cm containing sand (0–2 mm grain size, Hornbach, Hannover, Germany) in a climate chamber [22°C, 70% humidity, 12 h light/12 h dark, 480 μmol m^-2^ s^-1^ (lamp type CMT 360LS/W/BH-E40, Eye Lighting Europe Ltd, Uxbridge, UK)]. A total of 102 plants were grown, one plant per pot, for 19 d and watered once per week using 150 ml Blake-Kalff medium [22] containing 1 mM MgSO_4_. After a washing step with deionized water one half of the plants were transferred to “plus S” conditions with 1 mM MgSO_4_. The other half of the plants were transferred to “minus S” conditions using Blake-Kalff medium with only 10 μM MgSO_4._ Plants were grown under these conditions for 4 days. One hour before the light was switched on three plants of each treatment were then harvested every 4 h over a time period of 36 h. The material was pooled and immediately frozen in liquid nitrogen. Additionally 42 plants under “plus S” and “minus S” conditions were transferred to continuous light. These were then harvested at the same time as the plants grown under 12 h light/12 h dark every 4 h beginning after 16 h representing the beginning of the subjective night. For a 24 h cycle plants under continuous light were additionally harvested at 40 h. The complete experiment was performed twice.

### Sequence analysis

Sequences homologous to *Arabidopsis thaliana* DNA sequences for the genes *18S ribosomal RNA* (*18S rRNA)*, *Actin2 (ACT2)*, *ELONGATION FACTOR 1α (EF1α)*, *TATA BOX BINDING PROTEIN 2* (*TBP2*) and *TIP41-LIKE PROTEIN* (*TIP41)* as reference genes, as well as the *CIRCADIAN CLOCK ASSOCIATED1 (CCA1*) and *ADENOSINE 5’-PHOSPHOSULFATE REDUCTASE 3* (*APR3*) sequences as the GOIs were searched in the *B*. *napus* database (http://compbio.dfci.harvard.edu/compbio) [23] using BLAST. The data bank uses parts of short homologous sequences (high-fidelity virtual transcripts; TC-sequences, tentative consensus sequences) to generate EST sequences [24] that were used for primer design (http://www.dosbox.com) [25]. For the other reference genes *ADENINE PHOSPHORIBOSYL TRANSFERASE 1 (APT1)*, *GUANOSINE NUCLEOTIDE DIPHOSPHATE DISSOCIATION INHIBITOR 1 (GDI1)*, *SERINE/THREONINE PROTEIN PHOSPHATASE 2A (PP2A)*, and *UBIQUITINE-CONJUGATING ENZYME 21 (UBC21)* the database NCBI (https://blast.ncbi.nlm.nih.gov/Blast.cgi) was used due to the availability of *B*. *napus* sequences [26]. Primers were used to amplify cDNA fragments with a size of about 300 bp for Northern blot analysis and about 100 bp for RT-qPCR analysis (Table 1).

**Table 1:** Primer pairs used in this study.

Primer pairs	*A*.* thaliana* AGI	Sequences
P741_Bn_CCA1_for	At2g46830	5'-TTCTTGTGGCTCGAACACTCCT-3'
P742_Bn_CCA1_rev		5'-GGATTGGTGTTGCTGATGACTC-3'
P745_Bn_APR3_for	At4g21990	5'-CATCAAGGAGAACAGCAACGCA-3'
P746_Bn_APR3_rev		5'-TCGGGAACACTAGTATCGTCGG-3'
P747_Bn_EF1α_for	At5g60390	5'-GCTTGGTTGGAGTCATCTTCAC-3'
P748_Bn_EF1α_rev		5'-TCTCCTTGAGGCTCTTGACCAG-3'
P768A_Bn_TIP41_for	At4g34270	5'-GGCTTACGAATCCATGACTG-3'
P769B_Bn_TIP41_rev		5'-GAGGAGGAACCATGAACTTG-3'
P780_Bn_ACT2_for	At3g18780	5'-AACCTTCAACTCTCCAGCTA-3'
P781_Bn_ACT2_rev		5'-GAGTTGTAAGTCGTCTCGTG-3'
P782_Bn_18S rRNA_for	X16077.1	5'-ATGAACGAATTCAGACTGTG-3'
P783_Bn_18S rRNA_rev		5'-ACTCATTCCAATTACCAGAC-3'
P784_Bn_TBP2_for	At1g55520	5'-GGCTGAACAAGGAATGGAAG-3'
P785_Bn_TBP2_rev		5'-TCTCTCATCTTGGCTCCGGT-3'
P816_Bn_Act2_qPCR_f	At3g18780	5’-ACTCTCCAGCTATGTATGTCGCC-3’
P817_Bn_Act2_qPCR_r		5’-GAGACACACCATCACCAGAATCC-3’
P818_Bn_CCA1_qPCR_f	At2g46830	5’-GTCATCATCATCCTTGTGCAGCG-3’
P819_Bn_CCA1_qPCR_r		5’-GTGTTCGAGCCACAAGAAGACCT-3’
P822_Bn_APR3_qPCR_f	At4g21990	5’-AACGGCTAATGTCAATGGGACG-3’
P823_Bn_APR3_qPCR_r		5’-AAGCACAACGATCCAAGCCTCT-32019
P824_Bn_EF1α_qPCR_f	At5g60390	5'-GCAGATTGGTAACGGTTACG-3'
P825_Bn_EF1α_qPCR_r		5'-CTCCTTACCAGAACGCCTGT-3'
P960_Bn_q18SrRNA_f	X16077.1	5'-TGCAACAAACCCCGACTTCT-3'
P961_Bn_q18SrRNA_r		5'-TGCGATCCGTCGAGTTATCA-3'
P962_Bn_qTIP41_f	At4g34270	5'-GCGGCACGATTCTCACTTCT-3'
P963_Bn_qTIP41_r		5'-CACTAACGCATTCTCGCCAA-3'
P968_Bn_PP2A_f	At1g69960	5'-ACGAGGACGGATTTGGTTCC-3'
P969_Bn_PP2A_r		5'-GCTCCGAGCTTGTCATCGAA-3'
P970_Bn_qPP2A_f	At1g69960	5'-GTCAACAATCCGCACTACCTACA-3'
P971_Bn_qPP2A_r		5'-ACCACAGGAAGAAACTTAGAGCA-3'
P976_Bn_APT1_f	At1g27450	5'-TTCTTCTCGACACAGAGGCG-3'
P977_Bn_APT1_r		5'-TTCTCCCTGCCCTTAAGCTCT-3'
P978_Bn_qAPT1_f	At1g27450	5'-CATTGCTACGGGTGGGACTC-3'
P979_Bn_qAPT1_r		5'-CCCTTAAGCTCTGGTAACTCAATCA-3'
P980_Bn_UBC21_f	At5g25760	5'-ATCACGAGCGAGACTGTTCA-3'
P981_Bn_UBC21_r		5'-CCTCAGGATGAGCCATCAGT-3'
P982_Bn_qUBC21_f	At5g25760	5'-GACTGCACTTATCAAGGGACCG-3'
P983_Bn_qUBC21_r		5'-ACGGTTCGGGAACAGCGAAT-3'
P984_Bn_GDI1_f	At2g44100	5'-TGCACGTTTCCAAGGAGGTT-3'
P986_Bn_GDI1_r		5'-CGGTCTGAGGGTTGTCAGTC-3'
P987_Bn_qGDI1_f	At2g44100	5'-CGAGCCTGTCAACGAACCCA-3'
P988_Bn_qGDI1_r		5'-ATCCAGTTCCTTGCCGGTGA-3'

To identify homologous genes in *B*. *napus*, the known sequences from *A*. *thaliana* were blasted against the *B*. *napus* databases [23,26]. for: forward; rev: reverse; *CCA1*: *CIRCADIAN CLOCK ASSOCIATED1; APR3*: *ADENOSINE 5’ PHOSPHOSULFATE REDUCTASE 3; EF1α*: *ELONGATION FACTOR 1α; TIP41*: *TIP41-LIKE PROTEIN; ACT2*:*ACTIN2; 18S rRNA*: *18S RIBOSOMAL RNA; TBP2*: *TATA BOX BINDING PROTEIN 2; PP2A*: *SERINE/THREONINE PROTEIN PHOSPHATASE 2A; APT1*: *ADENINE PHOSPHORIBOSYL TRANSFERASE 1; UBC21*: *UBIQUITINE-CONJUGATING ENZYME 21; GDI1*: *GUANOSINE NUCLEOTIDE DIPHOSPHATE DISSOCIATION INHIBITOR 1*.

### RNA extraction and Northern blot analysis

Total RNA was extracted from ground plant material according to [27] and spectrophotometrically quantified. Fifteen μg RNA was separated on 1% denaturing agarose-formaldehyde gels. Equal loading was examined by staining the gels with ethidium bromide. After RNA transfer onto nylon membranes, they were probed with digoxigenin (DIG)-labeled cDNA probes obtained by PCR (PCR DIG probe synthesis kit, Roche, Mannheim, Germany). To amplify the respective probes, the sequence-specific primers listed in Table 1 were used. Colorimetric detection was performed using nitro blue tetrazolium (NBT) and 5-bromo-4-chloro-3-indolyl-phosphate (BCIP) as substrates for alkaline phosphatase. Quantitative analysis of the Northern blot results was done by GelAnalyzer 2010a (www.GelAnalyzer.com) [28].

### cDNA synthesis

Isolated RNA was spectrophotometrically quantified and afterwards, 250 ng of the total RNA was utilized for reverse transcription. As a first step, the remaining DNA was degraded by DNaseI (Thermo Fisher Scientific Inc., Waltham, USA). For the cDNA synthesis, oligo-(dT)_18_-primers from the First Strand cDNA Synthesis Kit (Thermo Fisher Scientific Inc.) were used. The addition of 1 μL RiboLock RNase inhibitor (Roche) enhanced the quality of the cDNA.

### Semi-quantitative qPCR

Semi-quantitative PCR (sqPCR) assays were performed with a Thermocycler cyclone 25 (PeqLab, Erlangen, Germany) using Dream *Taq*^TM^ DNA Polymerase (Thermo Fisher Scientific Inc.) and the primers listed in Table 1. For each primer pair, we determined the number of cycles with differently diluted cDNA where the amplification was still exponential. Products of sqPCR were separated on a 2% agarose gel and quantified by the intensity of the bands relative to the first band using the program GelAnalyzer 2010a [28].

### Quantitative real-time PCR

Quantitative real-time PCR (RT-qPCR) was realized with SYBR Green fluorescence and a ROX reference dye (Platinum SYBR Green RT-qPCR Mix; Thermo Fisher Scientific Inc.) on an ABI PRISM 7300 sequence detection system (Thermo Fisher Scientific Inc.) with the primers listed in Table 1. Raw data were converted into expression data by the ΔCt method [29].

### GrayNorm algorithm

Expression data of selected reference genes obtained by Northern blot analysis was evaluated with the GrayNorm algorithm according to [19] to maximize data accuracy by selecting the right combination of reference genes for each particular experiment.

### Statistical analysis

Six biological samples each with three technical replicates were used to determine the technical and biological variability. A restricted maximum likelihood (REML) variance component estimation in a linear mixed-effects model (for more information see [30]) was performed, where the technical variability within the biological replicates was determined by calculating the ratio of the biological to the technical variance component. Furthermore, the costs to perform each method per sample were individually calculated. Additionally data were compared in a Bland Altman Plot [31]. Using this method, agreements between individual measurements can be quantified whereas the correlation factor only measures the strength of a relation between two variables [32]. Each data point for every sample obtained using both methods is directly compared by calculating the difference and the average of each individual data point. The difference of each data point was then plotted against the average of each data point from both methods. Ideally, the data points lie very close to the mean of the difference indicating a high agreement between both methods. Analyzing the data obtained by both methods in a proper way the data were standardized first by centralizing the mean for both methods to zero and then divided by the standard deviation to achieve unit variance. The 95% limits of agreement were calculated with ±1.96xSD for the two or three technical replicates respectively ([29], chapter 5.2).

The relative expression with non-normalized and normalized data were evaluated using a Two-Way ANOVA with the relative band intensities as dependent variable and S concentration and time point of harvest as independent factors. For the independent factors S concentration, time point of harvest and light a Three-Way ANOVA was performed with the relative band intensities as dependent variable. Significance of factors and their interactions was assessed by means of F-tests.

## Results

### Comparison of the methods reveals the same trends of expression

To compare the technical variability of the three methods, an expression analysis of *ACT2* as a reference gene with a low biological variability was performed. Therefore, samples from plants grown under “plus S” conditions and 12 h light/12 h dark harvested every 4 h starting 1 h before the light was switched on were used. The total RNA was isolated out of three technical replicates for each sample and quantified. This RNA was then used as the initial point for all three methods.

Both Northern blot analysis and PCR-based methods showed a number of advantages and disadvantages. For Northern blot analysis, the isolated RNA was directly used for expression analysis, whereas for the PCR-based methods it was necessary to first perform reverse transcription. However, high amounts of RNA (15 μg per sample) were needed for Northern blot analysis. In contrast, 250 ng was sufficient to perform adequate cDNA synthesis. Evaluation of the success of cDNA synthesis was only possible by using control reactions, which were processed later on as samples in the PCR. For Northern blot analysis, the process was evaluated after the electrophoretic separation as well as after the blotting by visualizing the RNA under UV light. For the sqPCR pre-experiments, it was necessary to identify the correct cycle number and amount of template for every primer pair separately. On the other hand, these remained constant for each primer pair used in Northern blot analysis and RT-qPCR. The results obtained by Northern blot analysis and sqPCR are based on band intensities on the membrane or in the gel. Therefore, careful documentation was necessary as well as suitable software to measure band intensity. Inadequate membrane or gel quality can lead to false results that in turn provoke false biological conclusions. For RT-qPCR, quantification is more precise by measuring the amount of synthesized DNA in real-time. Nevertheless, all three methods are highly dependent on high-quality non-degraded RNA, precise pipetting to guarantee the same total amount of mRNA and a good documentation of results. Especially for the PCR-based methods, a number of steps during the process were dependent on precise pipetting indicated by less standard deviation after training, making this a big source of possible errors during sample preparation.

Based on the first results of *ACT2* expression analysis, sqPCR showed less agreement with the other two methods. Using sqPCR, the transcript level of *ACT2* was higher in the light phase, whereas its transcript levels were decreased in the light phase for Northern blot analysis and RT-qPCR (data not shown). Furthermore, the technical variability was about 44% higher than the biological variability (Table 2). With Northern blot the lowest technical variability of about 14% was shown. Investigating the suitable cycle number for the sqPCR was very time consuming and may vary for each primer pair used. Therefore, this method was excluded in further experiments.

**Table 2:** Overview of the costs, duration and technical to biological ratio of the methods.

	Costs (€ per sample)	Costs for 34 samples and 10 genes*	Duration (18 samples)	Technical:biolo-gical variation (%)
**Northern blot analysis**	2.01	~700	3 d	14.9
**Semi-quantitative PCR (sqPCR)**	3.29	~600	8 h	44.5
**RT-qPCR**	6.04	~1500	6 h	21.4

* The costs were calculated based on the whole data set consisting of 34 samples in total and 10 genes for expression analysis. For the calculation 3 repetitions per sample were taken into consideration. The cDNA synthesis for every sample was calculated for the PCR-based methods only once due to reuse of the cDNA for each primer system. The costs to perform the methods for one sample excluding RNA isolation were estimated in the same way for all three methods. Only the costs for the required materials were included in this calculation. Duration describes the time needed to perform each method after the RNA was isolated. For calculating the ratio between the biological to technical variation, a REML variance component estimation was performed.

**Fig 1:**
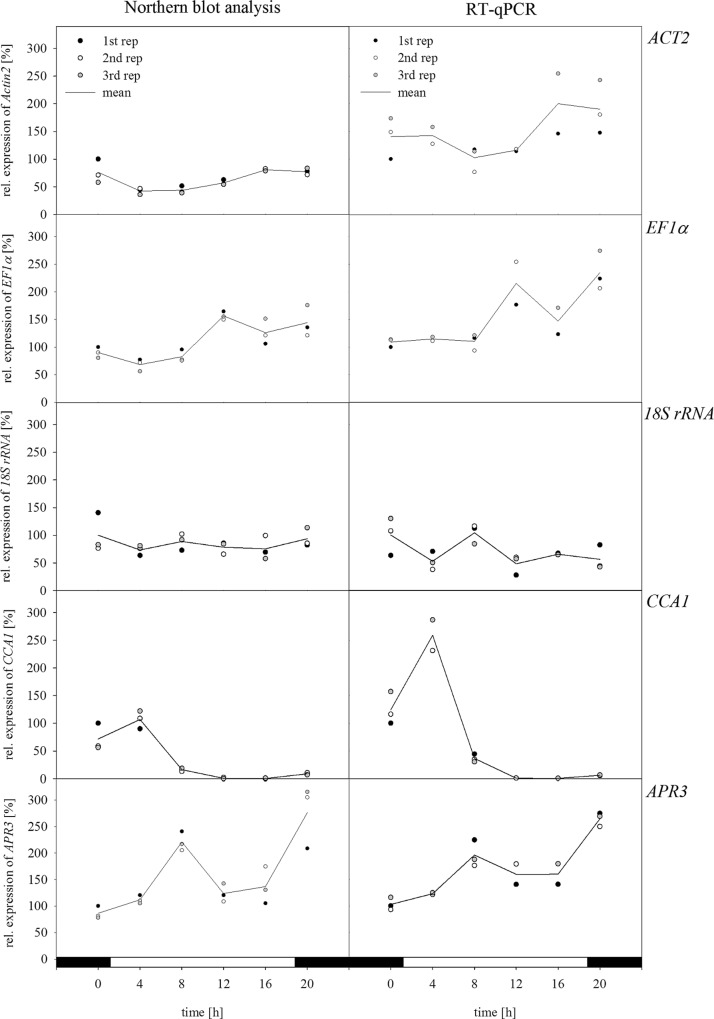
Comparison of Northern blot analysis and RT-qPCR analysis. Plants with five fully expanded leaves were harvested over a period of 20 h every 4 h, starting 1 h before the onset of light. Leaves from three plants were harvested and pooled. The relative expression for two to three technical replicates of each sample and the resulting mean is shown. Relative expression for Northern blot analysis was calculated based on the band intensity. Percentages refer to the first mean of the three technical replicates as 100% for both methods separately.

Northern blot and RT-qPCR analysis were performed to compare the expression of additional genes (Fig 1). Besides *ACT2*, *EF1α* and *18S rRNA* were chosen as other commonly used reference genes. In addition, one of the key genes in sulfate assimilation, *APR3*, and *CCA1* as part of the circadian oscillator were included in the expression analysis. The mean of two to three technical replicates showed the same trends of expression over the day for all five genes using both methods. However, the replicates of RT-qPCR analysis showed higher variations in their relative expression as compared to those in the Northern blot analysis. Moreover, some replicates for RT-qPCR analysis could not be used for the evaluation due to a missing fluorescence signal for these samples and were therefore omitted.

For *ACT2*, a higher expression level was measured 1 h before the light was switched on, as well as for plants harvested 1 h before the light was switched off as compared to the other time points (Fig 1). Interestingly, *ACT2* relative expression obtained by Northern blot analysis showed a decrease of about 30% after 3 h of light, which was not the case with RT-qPCR analysis. However, for both methods, lower *ACT2* transcript levels were observed in the middle of the light phase followed by an increase at the end of the light phase. Moreover, higher oscillations of the relative expression occurred due to high variations between the technical replicates when using RT-qPCR analysis.

As another reference gene, the expression of *EF1α* was analyzed (Fig 1). For both methods, a high degree of upregulation was observed in the middle of the light phase, remaining high at the end of the light phase and increasing slightly again at the beginning of the dark phase. Although the trend of expression was in agreement for both methods again high variations between the three replicates for RT-qPCR analysis were observed.

As a third reference gene the expression of *18S rRNA* was analyzed as well. First Northern blot analyses resulted in high band intensities indicating a possible saturated signal which might lead to underestimation of differences in the transcript amount. Therefore, the probe was tested for different RNA concentrations ranging from 1 to 24 μg revealing a saturated signal already at low RNA concentration. According to the results the *18S rRNA* probe was diluted 1:10 for further experiments (S1 Fig). Comparing the expression of *18S rRNA* with both methods higher oscillations for the RT-qPCR analysis were shown compared to the Northern blot analysis (Fig 1).

Relative expression levels of *APR3* showed for both methods an upregulation in the middle of the light phase as well as for the beginning of the night phase. Oscillations of transcript amounts were in this case higher when using Northern blot analysis (Fig 1). Based on its circadian regulation, *CCA1* showed a typical expression pattern during the course of the day. In the morning, before the light was switched on, relatively high *CCA1* transcript levels were detected. Three hours after the light was switched on, the highest degree of upregulation appeared. Afterwards, transcript levels decreased and reached almost undetectable levels at the end of the light phase. In the middle of the night phase, *CCA1* expression increased again. Both methods led to similar expression patterns. However, using RT-qPCR analysis, *CCA1* relative expression increased up to 150% compared to the first time point, whereas with Northern blot analysis expression was only 25% higher (Fig 1).

**Fig 2:**
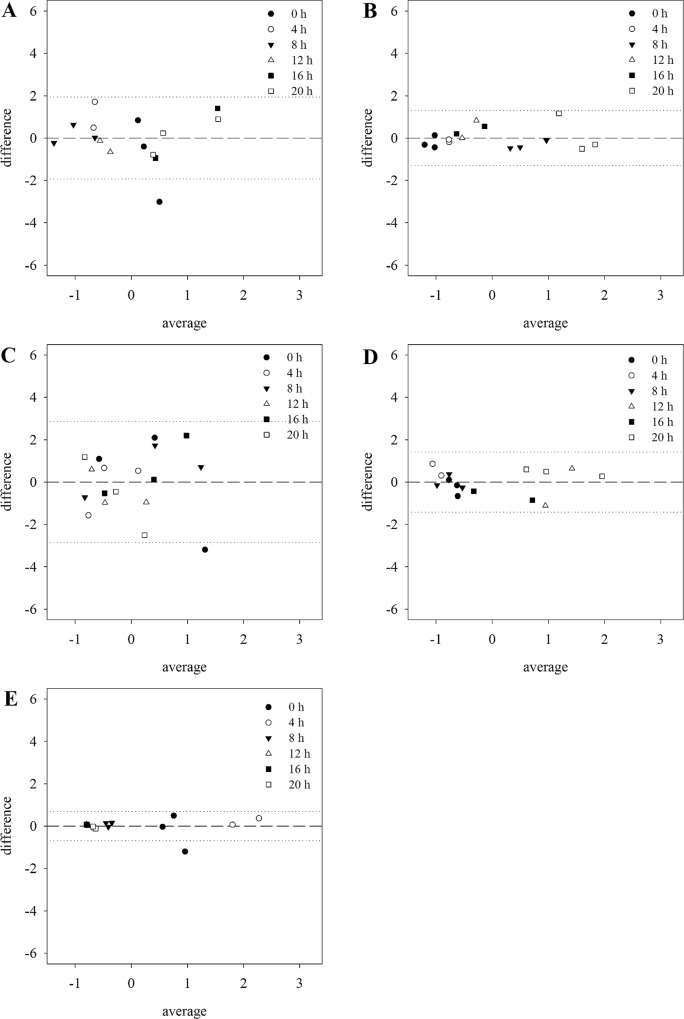
Bland Altman plot for method comparison. Data from Fig 1 were used to generate all graphs. For each data point from both methods, the value was standardized and the difference between the measurements by the two methods was plotted against their mean. Data are shown with the mean of the difference (dashed line) and the 95% limits of agreement ± 1.96xSD (dotted lines). A) *ACT2* B) *EF1α* C) *18S rRNA* D) *APR3* and E) *CCA1*.

Additionally, the methods were compared in a Bland Altman plot (Fig 2). For all genes, most of the two or three replicates for each harvesting time point were located around the mean of the difference. Furthermore, limits of agreement based on the standard deviation of the mean were added as well. Data points within these limits indicated a reliable agreement between both methods. Especially with *CCA1* expression analysis, most of the data points lie very close to zero. The lowest/worst agreement of the results was observed for *ACT2* and *18S rRNA*, indicated by higher variability around zero and wider limits of agreement.

Again all three methods were additionally compared by estimating the costs for one sample as well as for performing the methods with a big data set, working hours and the ratio of technical to biological variability (Table 2). The Northern blot analysis showed a low technical variability with about 14.9% and low costs per sample as well (Table 2). However, expression analysis by PCR-based methods is less time-consuming but results in much higher costs. The RT-qPCR analysis results revealed a lower technical variability than the sqPCR. Regarding the costs for performing these methods with a large sample set the RT-qPCR analysis would be the most expensive method to use. Taking the technical to biological variation and the costs into consideration, Northern blot analysis would be the method of choice for expression analysis with a large sample set. Moreover, comparison of the trend of expression of selected genes obtained by Northern blot and RT-qPCR as well as the comparison of both methods in a Bland Altman Plot supported the use of a traditional method as an alternative even more.

### Constitutive expression of reference genes should not be taken for granted

**Fig 3:**
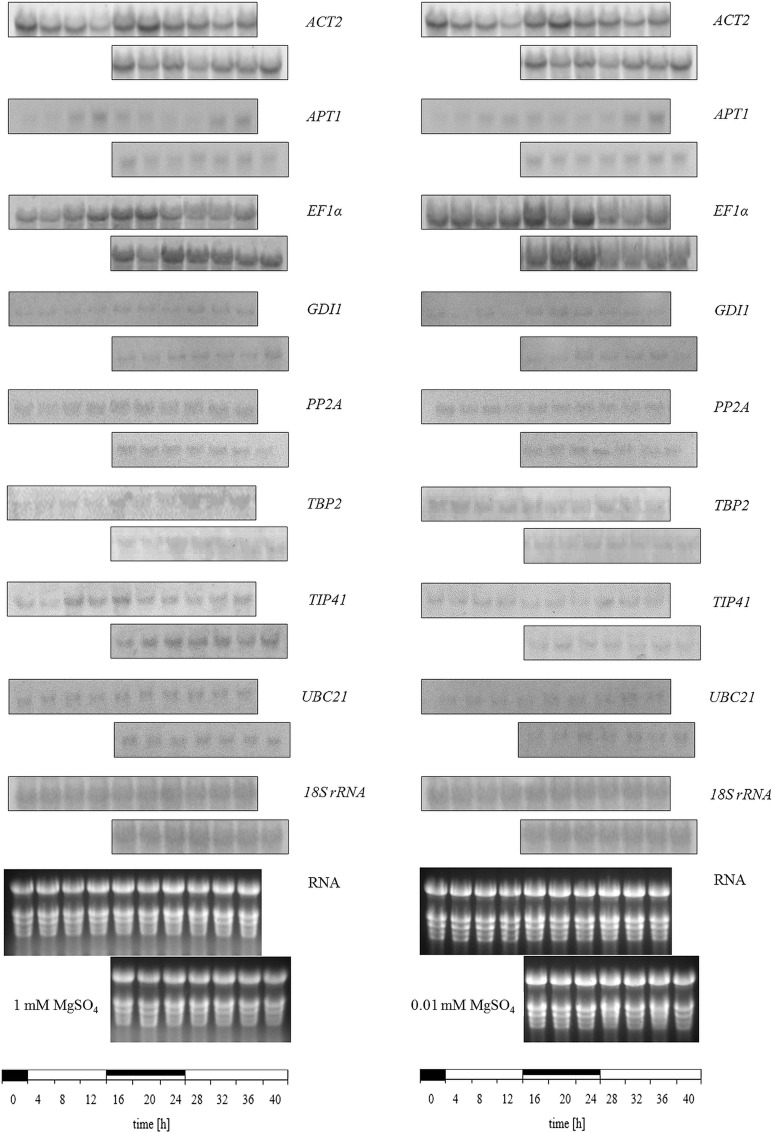
Expression of selected reference genes under circadian and S limiting conditions. Expression levels were analyzed in plants (with five fully expanded leaves) grown using 1 mM MgSO_4_ as a control and using 0.01 mM MgSO_4_ for four days to obtain S-limiting conditions. Plants were harvested over a period of 40 h every 4 h starting 1 h before the onset of light (left blot per gene). In addition, plants grown in a chamber with continuous light were harvested after 16 h (right blot per gene). Total RNA was isolated, and for Northern blot analysis 15 μg RNA was electrophoretically separated and transferred onto membranes. The detection of mRNA was done with probes labeled with DIG. Abbreviations: see legend of Table 1. The first row for the genes represents the 12 h light/12 h dark conditions and the second row represents the 24 h light conditions.

After choosing the method for a quantitative expression analysis the normalization had to be optimized under the given experimental conditions by finding suitable reference genes. To analyze the possible influence of the circadian rhythm on gene expression levels, plants were harvested every 4 h under diurnal and circadian conditions (Fig 3). Furthermore, half of the plants were grown under S deficiency to investigate a possible influence on gene expression as well. In this experiment, *ACT2*, *EF1α* and *18S rRNA* expression levels were analyzed again. However, first results showed an unstable expression of these commonly used genes. Therefore, the experiment was complemented with transcript levels of six additional reference genes *APT1*, *GDI1*, *PP2A*, *TBP2*, *TIP41*, and *UBC21* (Figs 2 and S2). The expression of *ACT2* was upregulated during the dark under diurnal conditions (Fig 2). This trend was unaffected by continuous light. Moreover, the expression was also unaffected by S-limiting conditions. On the contrary *APT1* showed a relatively constant expression level under continuous light whereas under diurnal conditions higher transcript levels were detected at the end of the light phase. The expression of *APT1* was not influenced by the sulfur limiting conditions. *GDI1* showed a relatively stable expression in the course of a day. However, under sulfur deficiency transcript levels showed more oscillations in the course of the day. Under constant light the expression of *GDI1* was unaffected. The transcript level of *EF1α* was affected differently under the given conditions. In plants grown under full sulfur supply and diurnal conditions *EF1α* was upregulated during the dark phase. Except for 20 h this was also the case in plants exposed to continuous light. However, here after the subjected night the degree of downregulation in the light phase was not as high as under diurnal conditions. In plants grown under sulfur-limiting conditions the expression of *EF1α* was regulated in different ways. Here under diurnal conditions transcript amounts were higher in the first light phase than with full sulfur supply. Moreover, expression of *EF1α* was unaffected after the subjective night compared to diurnal conditions. Furthermore, expression of *EF1α* was slightly upregulated under S-limiting conditions. Analysis of *PP2A* under the given conditions showed only slight oscillations in the course of the day were detected. Under the growth conditions the plants were grown under expression of *PP2A* was only influenced slightly. For the expression of *TIP41*, only slight differences in the transcript amount in the course of the day were detected. Under circadian conditions, the expression was at a more stable level. Sulfur-limiting conditions led to a slight down-regulation of *TIP41*. On the contrary, the reference gene *TBP2* was slightly upregulated under sulfur-limiting conditions. Moreover, a more constant transcript amount was measured in plants grown under sulfur-limiting conditions in the course of the day and expression was unaffected by the continuous light. Under sufficient sulfur supply only a low level of *TBP2* in the beginning of the light phase was detected increasing at the end of the light phase. For the second light phase higher transcript amounts were detected. In plants exposed to continuous light *TBP2* was upregulated and showed less oscillation in the transcript amount. Analyzing the expression of *UBC21* an unstable expression in the course of a day was shown which was further influenced under sulfur-limiting conditions. The expression of *18S rRNA* was influenced neither by the diurnal or circadian, nor by the S-limiting conditions. Except for *18S rRNA*, reference genes did not show steady expression levels under the given conditions. The two biological repetitions of the entire experiment revealed principally the same results within a variation less than 15%.

### Choosing the right reference genes is indispensable for a reliable expression analysis of the target genes

Expression data for the reference genes used in this study were analyzed by the GrayNorm algorithm [19] in consideration of the given plant growth conditions. Based on these results, a set of reference genes yielding the lowest level of uncertainty was validated for normalization purposes. In addition, single commonly used reference genes were chosen to compare the effect of normalization when using a set of reference genes and a single gene. Samples from plants grown under diurnal and circadian conditions were used for analyzing changes in the expression pattern and were measured for three dependent technical replicates.

**Fig 4:**
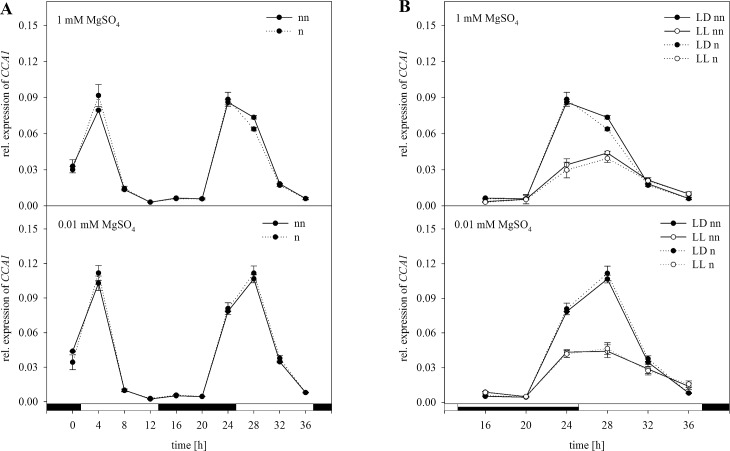
Normalization of *CCA1* expression with the validated set of reference genes *18S rRNA*, *PP2A*, and *GDI1*. Expression analysis for *CCA1* was performed in plants treated as previously described in Fig 3 by Northern blot analysis. Results were normalized using three reference genes according to Fig 3. The normalized (n) and non-normalized (nn) data are presented as the relative expression under (A) diurnal conditions over a period of 36 h and (B) free-running conditions with continuous light (LL) in comparison to the light-dark (LD) conditions. Data are shown as the mean of three technical replicates ± SD. Relative expression calculation was based on band intensity.

According to the GrayNorm analysis the lowest coefficient of variation can be achieved when using the combination of *18S rRNA*, *PP2A*, and *GDI1* for normalization. These results indicated that a set of three genes is sufficient for a proper normalization of the expression data for the GOIs. In this study the expression of *CCA1* and *APR3* was analyzed. The expression of the gene *CCA1* regulated in a circadian way was normalized with the validated set of reference genes (Fig 4). Without normalization, *CCA1* showed a high degree of upregulation in the morning followed by a downregulation in the course of the day, again followed by an upregulation in the morning (Fig 4A). Based on Two-way-ANOVA these oscillations were statistically significant. Under S limitation, the expression pattern was similar, although relative expression was significantly higher than in plants grown under full S supply in the beginning of the light phase. When normalized using the validated set of the reference genes *18S rRNA*, *PP2A*, and *GDI1*, the same trend of expression was observed. However, there was a higher expression of *CCA1* at 24 h when normalized. The two-way ANOVA of the non-normalized data revealed a significant effect of the harvesting time point and S status on the expression of *CCA1* (S1 Table). Moreover, there was a significant interaction between both parameters. Interestingly, for the normalized data, the effect of the S status on the expression of *CCA1* was not significant. However, there was a significant interaction between the S status and the point of time when analyzing the normalized data (S1 Table).

Additionally to plants grown in a light dark cycle, some plants were exposed to continuous light resulting in the same expression trend (Fig 4B). However, under continuous light the transcript amount of *CCA1* was decreased. There were no differences for the expression pattern when normalizing data with the validated set of reference genes. Including the third parameter light a three-way ANOVA was performed. The effect of light on the expression was significant whereas, the effect of S is independent of the light for non-normalized and normalized expression data (S2 Table). Moreover, ANOVA revealed a significant interaction between the time point and the light. Combining all three conditions the plants were grown under, there was a statistical interaction between these factors, which was independent of normalization.

**Fig 5:**
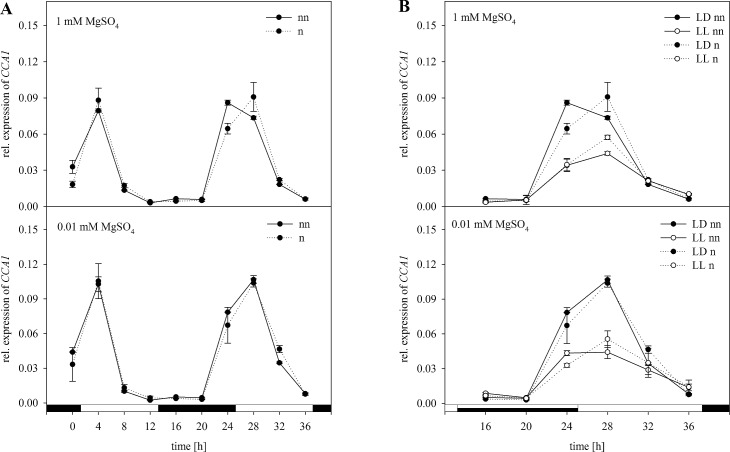
Normalization of *CCA1* with *ACT2*. Expression analysis for *CCA1* was performed in plants treated as previously described in Fig 3 by Northern blot analysis. Results were normalized using *ACT2* according to Fig 3. The normalized (n) and non-normalized (nn) data are presented as the relative expression under (A) diurnal conditions over a period of 36 h and (B) free running conditions with continuous light (LL) in comparison to the light dark (LD) conditions. Data are shown as the mean of three technical replicates ± SD. Relative expression calculation was based on band intensity.

For normalization with *ACT2* only, the expression pattern of *CCA1* compared to the non-normalized data remained the same (Fig 5). However, compared to the normalization with the set of reference genes the graphs are not as much in congruence especially when it comes to the continuous light (Fig 5B). In contrast to the set of reference genes there was a significant effect of the S status when analyzing the normalized data in a two-way ANOVA (S1 Table). However, the interaction between the S status and the time of harvesting was not significant. For the expression data in plants exposed to continuous light (Fig 5B), normalization with *ACT2* led to the same expression trend. However, there was a difference in the results of three-way ANOVA. When normalizing with *ACT2*, there was no significant interaction of the three factors S status, light and time.

**Fig 6:**
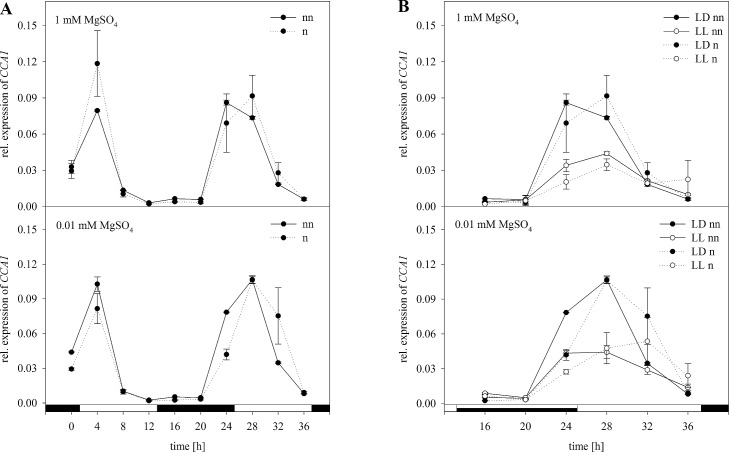
Normalization of *CCA1* with *EF1α*. Expression analysis for *CCA1* was performed in plants treated as previously described in Fig 3 by Northern blot analysis. Results were normalized using *EF1α* according to Fig 3. The normalized (n) and non-normalized (nn) data are presented as the relative expression under (A) diurnal conditions over a period of 36 h and (B) free running conditions with continuous light (LL) in comparison to the light dark (LD) conditions. Data are shown as the mean of three technical replicates ± SD. Relative expression calculation was based on band intensity.

As *EF1α* was most affected under the experimental conditions, normalization with it was performed as well (Fig 6). Comparing the resulting graphs to non-normalized data there were a number of disagreements. Interestingly, for normalization with *EF1α* the relative expression at 4 h under full S supply was about one third higher than for the non-normalized data (Fig 6A). Furthermore, under constant free-running conditions normalization with *EF1α* led to a lower level of expression in the morning. Under S-limiting and diurnal conditions, in contrast the amplitude was lower at 0 and 4 h after normalization. Furthermore, the relative expression of *CCA1* at 24 h was nearly 50% lower in comparison to the non-normalized data. Whereas after 32 h normalization led to a doubling of the expression level compared to non-normalized data. Under free-running conditions the expression level at 32 h was nearly the same as for 28 h when normalizing with *EF1α* (Fig 6B). The two-way ANOVA revealed a significant interaction between the S status and the time point of harvest for the non-normalized as well for the normalized data (S1 Table). There was no significant difference between normalizing with *EF1α* or the suggested set of reference genes. Including the light factor and performing a three-way ANOVA there is a significant interaction between the S status, the time of harvesting and the light condition when analyzing the non-normalized data (S2 Table). Normalizing with the suggested set of reference genes led to the same result for statistical analysis. However, normalized data with *EF1α* showed a non-significant interaction between these three factors.

**Fig 7:**
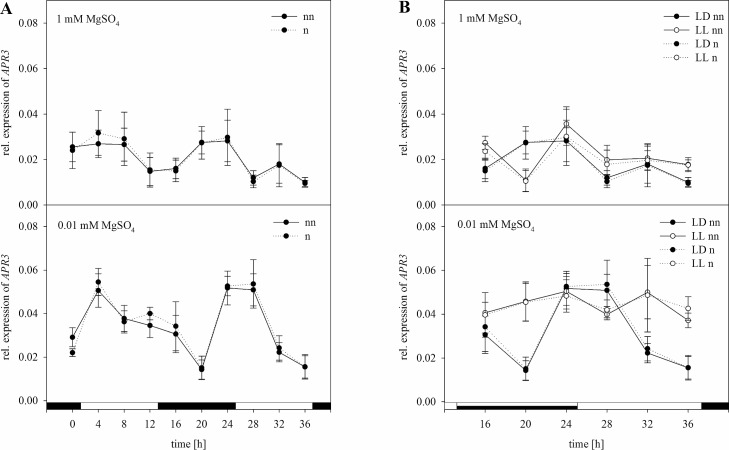
Normalization of *APR3* with the validated set of reference genes *18S rRNA*, *PP2A*, and *GDI1*. Expression analysis for *APR3* was performed in plants treated as previously described in Fig 3 by Northern blot analysis. Results were normalized using three reference genes according to Fig 3. The normalized (n) and non-normalized (nn) data are presented as the relative expression under (A) diurnal conditions over a period of 36 h and (B) free running conditions with continuous light (LL) in comparison to the light dark (LD) conditions. Data are shown as the mean of three technical replicates ± SD. Relative expression calculation was based on band intensity.

The relative expression of *APR3* as part of the S assimilation pathway was normalized as well (Fig 7). In plants grown under full S supply, transcript levels of *APR3* were significantly higher in the middle of the light phase as well as after 16 h at the end of the night phase (Fig 7A). In plants grown under S-limiting conditions, *APR3* was upregulated only after 20 h again. Moreover, a significantly higher upregulation was shown under S deficiency. A significant interaction between the S status and the harvesting time of the plants was revealed by performing a two-way ANOVA (S3 Table). Normalizing the expression data with the set of reference genes had no effect on the expression trend. However, there was a higher upregulation in the beginning of the first light phase and in the end of the dark phase with 1 mM MgSO_4_. Under S-limiting conditions, a slightly lower degree of upregulation was observed. However, statistical analysis of the normalized data revealed the same results as for non-normalized data. In plants exposed to continuous light, upregulation started at the end of the subjective night and was kept at a nearly steady transcript level (Fig 7B). Under S-limiting conditions, a significantly higher degree of *APR3* upregulation was measured at the beginning of the light phase. Interestingly, under free-running conditions, no oscillations for the relative expression could be determined. For the non-normalized as well as for the normalized data, there was a significant interaction in the three-way ANOVA between the S status and light (S4 Table). Interestingly, no significant interaction was observed between the time and light without normalization, whereas analyzing the normalized data showed a significant interaction. In agreement with the non-normalized data, the combination of all three factors showed a significant interaction when normalized using the validated set of reference genes.

**Fig 8:**
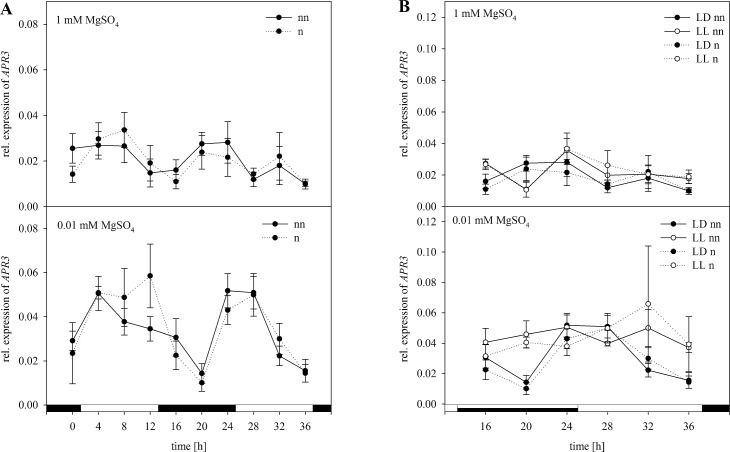
Normalization of *APR3* with *ACT2*. Expression analysis for *APR3* was performed in plants treated as previously described in Fig 3 by Northern blot analysis. Results were normalized using *ACT2* according to Fig 3. The normalized (n) and non-normalized (nn) data are presented as the relative expression under (A) diurnal conditions over a period of 36 h and (B) free running conditions with continuous light (LL) in comparison to the light dark (LD) conditions. Data are shown as the mean of three technical replicates ± SD. Relative expression calculation was based on band intensity.

Normalization of the *APR3* expression with *ACT2* as a single reference resulted in a different shape of the curve compared to the non-normalized data (Fig 8). Here a higher degree of upregulation in the light phase and a lower degree of upregulation in the night phase after normalization was shown (Fig 8A). Under S-limiting conditions, *APR3* expression was about one third higher than non-normalized expression data at 12 h. Despite the deviations in the results, they were similar to the normalized data with the set of reference genes after statistical analysis (S3 Table). Under free-running conditions (Fig 8B), there was a lower expression level in the subjective night and a higher expression level in the light phase at 28 h. Results of statistical analysis with the normalized data using *ACT2* were with one exception comparable to the normalized data with the set of reference gene (S4 Table).

**Fig 9:**
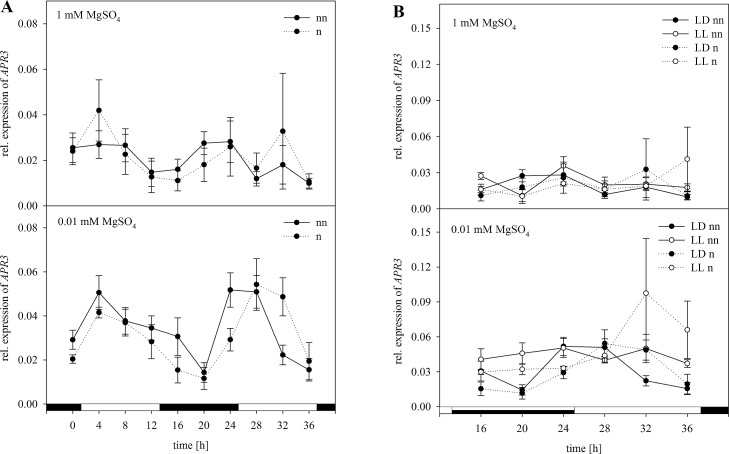
Normalization of *APR3* with *EF1α*. Expression analysis for *APR3* was performed in plants treated as previously described in Fig 3 by Northern blot analysis. Results were normalized using *EF1α* according to Fig 3. The normalized (n) and non-normalized (nn) data are presented as the relative expression under (A) diurnal conditions over a period of 36 h and (B) free running conditions with continuous light (LL) in comparison to the light dark (LD) conditions. Data are shown as the mean of three technical replicates ±SD. Relative expression calculation was based on band intensity.

Using *EF1α* as a reference gene more differences in the expression level after normalization occurred (Fig 9). Under full sulfur supply and diurnal conditions expression of *APR3* was nearly one third higher three hours after onset of light compared to the non-normalized data (Fig 9A). Furthermore, here the expression was slightly lower in the night and higher again in the middle of the day. Under sulfur-limiting conditions the expression level of the normalized data was slightly decreased for the first day and slightly increased for the second day compared to non-normalized data. Furthermore, normalization led to a shift of the expression maxima from 24 and 28 h to 28 and 32 h. Normalizing the expression data obtained under free running conditions led to major differences compared to the non-normalized data (Fig 9B). In the subjective night a much lower expression level was shown. Furthermore the expression level was nearly doubled after 32 h and 36 h. Statistical analysis showed that there is a significant interaction between the sulfur status and the time point of harvest for the non-normalized data (S3 Table). This was also the case when normalizing with the suggested best choice. However, analyzing the *EF1α* normalized data there was no significant interaction between these two factors. When it comes to factor light (S4 Table), there was as well a significant interaction between the three factors when analyzing the non-normalized data. In agreement with the suggested best choice this was also the case whereas normalized data with EF1α showed no significant interaction between the three factors.

To summarize, the expression pattern of *CCA1* and *APR3* certainly remained the same when normalizing with the validated set of reference genes, or with *ACT2* or *EF1α* instead. Nevertheless, normalization with a single reference gene led to greater discrepancy between the curves compared to normalization with the set of reference genes, where the curves were more similar. Moreover, analysis based on single reference genes resulted in large differences in expression values among sampling points after normalization.

## Discussion

Despite the development of newer methods, such as quantitative (real-time) PCR, nuclease protection assays, microarrays, and RNA-Seq, Northern blot analysis is still a standard technique used in the detection and quantification of mRNA [33,34]. There are a number of studies where Northern blot analysis and PCR-based methods were compared to assess if different methods affect the outcome [35–37]. Furthermore, it was suggested to verify gene expression by different methods whenever possible [36]. Regarding expression analysis in our study, we compared Northern blot analysis, sqPCR and RT-qPCR to find a suitable method for our experimental setup involving three technical replicates of selected samples. Based on the first results of analyzing the expression of *ACT2*, sqPCR was omitted due to a high technical variability within the biological replicates. With the two remaining methods, further experiments were performed to verify the reliability of the results. If results generated with Northern blot analysis were similar to those obtained with RT-qPCR, the reliability of both methods was confirmed. This was verified by comparing the trend of expression of selected genes by means of Northern blot and RT-qPCR analysis (Fig 1). This was also observed in previous studies [35–37]. However, in the end, RT-qPCR remained the method of choice in these studies due to its high sensitivity and speed. Despite such advantages, various technical parameters like pipetting and efficient reverse transcriptase can influence the accuracy and precision of the results during RT-qPCR [19].

In addition to comparing the trend of expression, each sample of each technical replicate was compared between the two methods in a Bland Altman Plot. In our study, nearly all data points lay within the 95% limits of agreement indicating an agreement between the measurements obtained from the two methods (Fig 2).

Taking the high sample amount of this complex experiment into consideration using RT-qPCR analysis would yield in higher costs compared to Northern blot analysis (Table 2). Based on these results, we selected Northern blot analysis to perform further experiments due to its simplicity, low costs, direct visibility and sufficient sensitivity for our purposes. However, the reliability of Northern blot analysis in a quantitative way needed to be further improved by optimizing the normalization with suitable reference genes.

Although they are commonly used as reference genes, neither *ACT2* nor *EF1*α showed a steady expression over the day, which makes their only use as reference genes inappropriate whereas the third commonly used reference *18S rRNA* showed only slight oscillations in the course of the day. However, using only one reference gene for normalization would be inappropriate. In further experiments, additional conditions like nutrition (S)-induced stress, continuous light and the time points of harvesting might also influence the expression of these reference genes. Therefore, we also analyzed six additional reference genes by Northern blot analysis under the conditions mentioned above. As a typical reference gene used for expression analysis, *ACT2* is present and constitutively expressed in all vegetative tissues in *A*. *thaliana* [38]. However, under abiotic stress like salt and cold, *ACT2* was not stably expressed anymore [13]. In our study (Fig 3), its expression was slightly reduced under S-limiting conditions. Moreover, in the course of a day oscillations of the transcript level of *ACT2* were detected.

According to literature *APT1* was stably expressed under a number of abiotic stresses [39]. In agreement with our results same transcript amounts were detected in plants grown under sulfur deficiency. However, expression was strongly affected under diurnal and circadian conditions due to its role in the cytokinin metabolic processes [40].

As part of the protein synthesis *EF1α* is also a commonly used reference gene, but its transcript amounts oscillated over the course of a day as well. Its expression opposed to *ACT2* was upregulated in plants grown under S-limiting conditions. In *B*. *napus* the expression of *EF1α* was affected in different directions under abiotic stress and only showed a stable expression under heavy metal stress [15].

As a novel reference gene *GDI1*, which is part of the membrane vesicular traffic, was validated as a stable expressed gene in *B*. *napus* under a number of stress conditions [41]. This is in agreement with our study when analyzing the expression in plants grown under diurnal or circadian conditions where the transcript amount was nearly not affected. However, higher oscillations occurred under these conditions when plants were exposed to sulfur limiting conditions.

In a study where reference genes were validated for a diurnal time course in lettuce the enzyme PP2A that removes phosphatase groups from the given substrate showed a stable expression. In agreement with our results under diurnal conditions *PP2A* showed in *B*. *napus* a relatively stable expression [42]. Furthermore the expression of *PP2A* analyzed under various abiotic stresses in *B*. *napus* could be determined as stably expressed for a number of treatments such as drought and salt stress [15]. Based on our results a constant expression is provided under sulfur limiting conditions as well. Even in plants exposed to continuous light the transcript amount of *PP2A* was nearly unaffected making it a promising candidate for a suitable reference.

The gene coding for the membrane protein channel TIP41 is recommended as an internal control due to a very stable expression in vegetative samples [43,44] and *TIP41* was stably expressed under biotic and abiotic stress as well [15]. In our study however, transcript levels of *TIP41* fluctuated in the course of the day. Interestingly, in lettuce *TIP41* was the most stably expressed reference gene under diurnal conditions [42]. Nevertheless fluctuations were not as high as for *ACT2* and *EF1α*. Furthermore the expression was only slightly down-regulated under S-limiting conditions.

As a non-traditional reference gene, expression of a gene encoding for a TATA box binding protein (*TBP2)* was analyzed as well. It was recommended as a reference gene because of its function as a transcription factor that binds DNA and therefore has a steady state level of expression [45]. In this study, transcript levels of *TBP2* only showed slight oscillations in the course of the day. Expression seemed to be influenced by the S status and light as well.

In previous studies a stable expression of *UBC21* in *B*. *napus* under various stress conditions was already verified [15,44]. According to our analysis in this study transcript amounts were indeed affected by the sulfur limiting conditions plants were grown under. Furthermore expression was influenced under diurnal and circadian conditions.

The use of 1*8S* and *28S rRNA* as an internal standard for expression analysis was recommended in 1999 by Thellin and colleagues [46], and *18S rRNA* was commonly used as a reference for expression analysis [47–49]. However, the use of *18S rRNA* as an internal control was often criticized due to its high transcript abundance compared to the GOI. Furthermore, for a number of plant species it was not recommended as a suitable reference gene (reviewed in [50]). Nevertheless, based on our results for our experimental design *18S rRNA* was the most stably expressed reference gene in *B*. *napus* among the other tested reference genes.

In agreement with our results, recent studies revealed that continuous expression under biotic and abiotic stress is not always provided and strongly depends on the used reference genes and experimental setup [13,15]. However, in these studies, plants were always exposed to one condition to reveal the best reference gene. In our study, the challenge was to analyze selected reference genes under combined experimental conditions such as the S status, time point of harvesting and free-running conditions like 24 h of light. According to our results of the Northern blot analysis *18S rRNA* would be the reference gene of choice. However, normalizing the expression level of a target gene with only one of the reference genes tested in this study would be inappropriate. Therefore, by using the GrayNorm algorithm, a combination of reference genes yielding the lowest level of uncertainty can be determined, ensuring a more reliable normalization [19]. This algorithm was actually developed evaluating RT-qPCR data. In this study data obtained by Northern blot analysis based on band intensities was used for GrayNorm analysis verifying suitable references confirming a proper normalization of the GOIs. This is actually the first time the GrayNorm algorithm was used for evaluating data generated by Northern Blot analysis. Regarding our study, the combination of *18S rRNA*, *PP2A*, and *GDI1* was verified as the best combination of reference genes with the lowest CV. This result indicates that normalization with three references is sufficient for normalization and also verifies the stability of the references in their expression under the given circumstances. In agreement with our results obtained by Northern blot analysis, these were the genes that were least affected in their expression. Demonstrating how the outcome was affected for our experimental setup, expression data based on Northern blot analysis were normalized with the validated set of reference genes and compared to normalization with single reference genes. Therefore, expression levels of *CCA1* and *APR3* were analyzed under the same conditions as described before (Figs 4–9). To demonstrate the effect of normalization, two- and three-way ANOVAs were performed to determine the influence of S status, time of harvest and light and possible interactions between the factors using non-normalized and normalized data respectively (S1–S4 Tables). For *CCA1*, normalization with the validated set of reference genes resulted in nearly the same trend of expression as compared to non-normalized expression data (Fig 4). However, normalized data revealed no significant effect of the S status on the expression of *CCA1* as opposed to non-normalized data (S1 Table). Based on these results, one would reason that the S status in the plants did not affect the circadian rhythm. In contrast, there was a significant influence of the S status on *CCA1* expression when non-normalized and normalized data with *ACT2* were considered (Fig 5, S1 Table). Whereas it is known that *CCA1* indirectly affects the nutrition metabolism [51], the opposite effect is not yet clearly demonstrated. Therefore, our results need to be interpreted with caution. Furthermore, as for the normalized data with the set of reference genes, three-way ANOVA revealed a significant interaction of the three conditions affecting the expression of *CCA1* (S2 Table). In contrast, there was no significant interaction when normalizing with *ACT2* or *EF1α* alone. Our results demonstrated clearly how normalization with only one reference gene can distort/invalidate the outcome and lead to false conclusions.

Normalizing *APR3* with *ACT2* or *EF1α* also led to disagreements compared to normalization with the set of reference genes (Figs 8 and 9). Here normalization with *ACT2* or *EF1α* seemed to have a stronger influence on the trend of expression compared to normalizing *CCA1*. However, within the analysis of *APR3* expression, investigating the influence of S and the time point of harvest, there was a clear interaction between both variables according to normalization with a suitable set of reference genes or *ACT2* whereas this was not significant when normalizing with *EF1α* (S3 Table). Furthermore when the third factor continuous light was included, the interaction between all three factors was highly significant for normalization with the selection of reference genes (p<0.001), *ACT2* alone (p<0.05), but not significant when normalizing with *EF1α* alone (S4 Table). According to our results, using only *ACT2* or *EF1α* as a reference gene would prevent a proper evaluation of the expression analysis. This clearly shows how important a proper validation of the used reference gene is before normalizing, even when those genes are commonly used reference genes such as *ACT2* or *EF1α*.

There are no universal reference genes in plants known so far with a constant transcript amount under different conditions across all plant species [12,52,53]. Therefore, a proper evaluation of references for new experimental set-ups or plants is inevitable for generating results one can rely on.

## Conclusion

In this study, it was confirmed that under several conditions like the S status in plants, the harvesting time point as well as light regime the expression of commonly selected reference genes can be strongly influenced. Using only one of them as a single reference gene to quantify the relative expression of GOIs under the circumstances used in this study, normalization would be inappropriate whereas a combination out of these genes determined by the GrayNorm algorithm seems to be the best choice. Whereas RT-qPCR is the standard to perform gene expression analysis, our results demonstrate that similar results can be obtained using Northern Blot analysis. However, also here a set of suitable reference is essential to guarantee a confident evaluation of gene expression analysis. To our best knowledge, this is the first time that data obtained with Northern blot analysis were normalized with a set of reference genes maximizing the accuracy of normalization and hence data interpretation in a complex experimental set up.

## Supporting Information

S1 Fig Northern blot analysis of *18S rRNA* with different RNA concentrations. Different concentrations (1 to 24 μg) of pooled RNA samples as described in the legend to Fig 1 were electrophoretically separated on a 1% agarose gel. The probe of *18S rRNA* was also diluted 1:10 before hybridization (in the middle).
(TIF)

S2 Fig Comparison of Northern blot analysis and RT-qPCR analysis for other tested reference genes. Plants with five fully expanded leaves were harvested over a period of 20 h every 4 h, starting 1 h before the onset of light. Leaves from three plants were harvested and pooled. The relative expression for two to three technical replicates of each sample and the resulting mean are shown. Relative expression for Northern blot analysis was calculated based on the band intensity. Percentages refer to the first mean of the three technical replicates set at 100% for both methods separately.
(TIF)

S1 Table Two-way ANOVA analysis of the expression data obtained for *CCA1*. (DOCX)

S2 Table Three-way ANOVA analysis of the expression data obtained for *CCA1*. (DOCX)

S3 Table Two-way ANOVA analysis of the expression data obtained for *APR3*. (DOCX)

S4 Table Three-way ANOVA analysis of the expression data obtained for *APR3*. (DOCX)
