# Supplementary material for: Quantitative Expression Analysis in Brassica napus by Northern Blot Analysis and Reverse Transcription-Quantitative PCR in a Complex Experimental Setting
Source: PLoS One. 2016 Sep 29;11(9):e0163679. doi: 10.1371/journal.pone.0163679 (PMC5042561; doi:10.1371/journal.pone.0163679)
Supplement: S1 Table — (DOCX) [file pone.0163679.s003.docx]

**S1 Table: Two-way ANOVA analysis of the expression data obtained for *CCA1*.**

|  | **p-value** | | | |
| --- | --- | --- | --- | --- |
|  | | **Sulfur status (S)** | **Time point of harvest (T)** | **SxT** |
| Non-normalized | | <0.001 | <0.001 | <0.001 |
| Set of reference genes | | <0.001 | <0.001 | <0.001 |
| *ACT2* | | 0.004 | <0.001 | 0.075 |
| *EF1α* | | 0.950 | <0.001 | 0.001 |
