# Supplementary material for: Quantitative Expression Analysis in Brassica napus by Northern Blot Analysis and Reverse Transcription-Quantitative PCR in a Complex Experimental Setting
Source: PLoS One. 2016 Sep 29;11(9):e0163679. doi: 10.1371/journal.pone.0163679 (PMC5042561; doi:10.1371/journal.pone.0163679)
Supplement: S3 Table — (DOCX) [file pone.0163679.s005.docx]

**S3 Table: Two-way ANOVA analysis of the expression data obtained for *APR3*.**

|  | **p-value** | | | |
| --- | --- | --- | --- | --- |
|  | | **Sulfur status (S)** | **Time point of harvest (T)** | **SxT** |
| Non-normalized | | <0.001 | <0.001 | <0.001 |
| Set of reference genes | | <0.001 | <0.001 | <0.001 |
| *ACT2* | | <0.001 | <0.001 | 0.002 |
| *EF1α* | | 0.005 | <0.001 | 0.085 |
