# Supplementary material for: Quantitative Expression Analysis in Brassica napus by Northern Blot Analysis and Reverse Transcription-Quantitative PCR in a Complex Experimental Setting
Source: PLoS One. 2016 Sep 29;11(9):e0163679. doi: 10.1371/journal.pone.0163679 (PMC5042561; doi:10.1371/journal.pone.0163679)
Supplement: S4 Table — (DOCX) [file pone.0163679.s006.docx]

**S4 Table: Three-way ANOVA analysis of the expression data obtained for *APR3*.**

|  |  | | **p-value** | | | | | | |
| --- | --- | --- | --- | --- | --- | --- | --- | --- | --- |
|  | | **Sulfur status (S)** | | **Time point of harvest (T)** | **Light** | **SxL** | **SxT** | **LxT** | **SxLxT** |
| Non-normalized | | <0.001 | | <0.001 | <0.001 | 0.014 | 0.078 | 0.089 | <0.001 |
| Set of reference genes | | <0.001 | | 0.003 | <0.001 | 0.023 | 0.089 | 0.070 | <0.001 |
| *ACT2* | | <0.001 | | 0.006 | <0.001 | 0.117 | 0.166 | 0.773 | 0.014 |
| *EF1α* | | <0.001 | | <0.001 | 0.01 | 0.026 | 0.037 | 0.054 | 0.221 |
